# The complete mitochondrial genome of *Propsilocerus akamusi* (Diptera, Chironomidae)

**DOI:** 10.1080/23802359.2019.1688703

**Published:** 2019-11-12

**Authors:** Qingji Zhang, Wenbin Xu, Kai Peng, Lianghua Zou, Ying Li, Ye Chen, Yongjiu Cai, Zhijun Gong

**Affiliations:** a State key laboratory of Lake Science and Environment, Nanjing Institute of Geography and Limnology, Chinese Academy of Science, Nanjing, People’s Republic of China;; b School of Marine Sciences, Ningbo University, Ningbo, People’s Republic of China

**Keywords:** *Propsilocerus akamusi*, Chironomidae, mitochondrial genome

## Abstract

*Propsilocerus akamusi* is one of the most important genera of Chironomidae. However, the systemically classification and taxonomic studies have so far been limited. In this study, we report the complete mitochondrial genome sequence of *P*. *akamusi*. The mitogenome has 15,632 base pairs (77.58% A + T content) and made up of total of 37 genes (13 protein-coding, 22 transfer RNAs, and 2 ribosomal RNAs). This study will provide useful genetic information for future phylogenetic and taxonomic classification of Chironomidae.

The species of the family Chironomidae account for about 25% of the freshwater lake biota, and its larval biomass accounts for 70–80% of the total number of benthic animals. *Propsilocerus akamusi* belongs to the Family Chironomidae and the Order Diptera. Because the Chironomidae are relatively large, easy to obtain, and diverse in life regional strong, benthic life and so on, so it is an excellent indicator organism for monitoring the water environment and pollution status (Saether 1977).

There is no report of the complete genome of this species *P. akamusi*, which was developed in Nanjing, Jiangsu Province, Republic of China (N32°07′31″, E118°81′01″) in September 2019. Therefore, it is very important to characterize the complete mitogenome of this species, which can be utilized in research on taxonomic resolution, population genetic structure and phylogeography, and phylogenetic relationship. Total DNA was extracted from *P. akamusi* individuality following TIANamp Marine Animals DNA Kit (Tiangen, China) and NOVOPlasty software was used to assemble the mitogenomes, the mistake parameter was set by default (Dierckxsens et al. 2017). The samples were stored at −80 °C in State key laboratory of Lake Science and Environment, Nanjing Institute of Geography and Limnology, Chinese Academy of Science, Nanjing, China. Number is PA-1.

In this study, we obtained the complete mitochondrial genome of the *P. akamusi*. Its mitochondrial genome has been deposited in the GenBank under accession number MN566452. For a better understanding of the genetic status and the evolutionary study, we focused on the genetic information contained in the complete mitochondrial genomes of the Chironomidae.

The complete mitogenome of the *P. akamusi* was 15,632 bp in length. The genomic organization was identical to those of typical Diptera mitochondrial genomes, including 2 rRNA genes, 13 protein-coding genes, and 22 tRNA genes. The overall base composition was 39.7% of A, 37.9% of T, 9.8% of C, and 12.6% of G with a slight A + T bias (77.6%) like other Chironomidae mitochondrial genomes. The features mentioned above were accordant with typical Chironomidae mitogenome.

For the 13 protein-coding genes, 6 genes (*Cytb*, *ND4L*, *ND4*, *COII*, *COIIIi,* and *ATP6*) started with ATG, 5 genes (*ND2, ND3, ND5 ND6*, *and ATP8*) started with ATT and *COI and ND1* started with TTG. All 13 genes shared the termination codon TAA This feature was common among Chironomidae mitochondrial protein-coding genes. The complete mitogenome sequence had s-rRNA (801 bp) and l-rRNA (1338 bp), which were separated by trnV. The location is same as most Chironomidaes that have high conservation.

To determine the taxonomic status of *P. akamusi*, we reconstructed the phylogeny of this species with other natural populations based on the *COI* gene. The phylogenetic tree showed that the *P. akamusi* has a closer relationship with *Phaenopsectra flavipes, Polypedilum quadriguttatum, Pontomyia natans*, *and Virgatanytarsus aboensi* (Figure 1). The phylogeny was reconstructed based on the General Time Reversible + Invariant + gamma sites (GTR + I+G) model of nucleotide substitution using Mega version 7 (PA, USA) (Kumar et al. 2016). The complete mitochondrial genome sequence of the *P. akamusi* provided an important dataset for a better understanding of the mitogenomic diversities and evolution in Chironomidae as well as novel genetic markers for studying population genetics and species identification.

**Figure 1.**
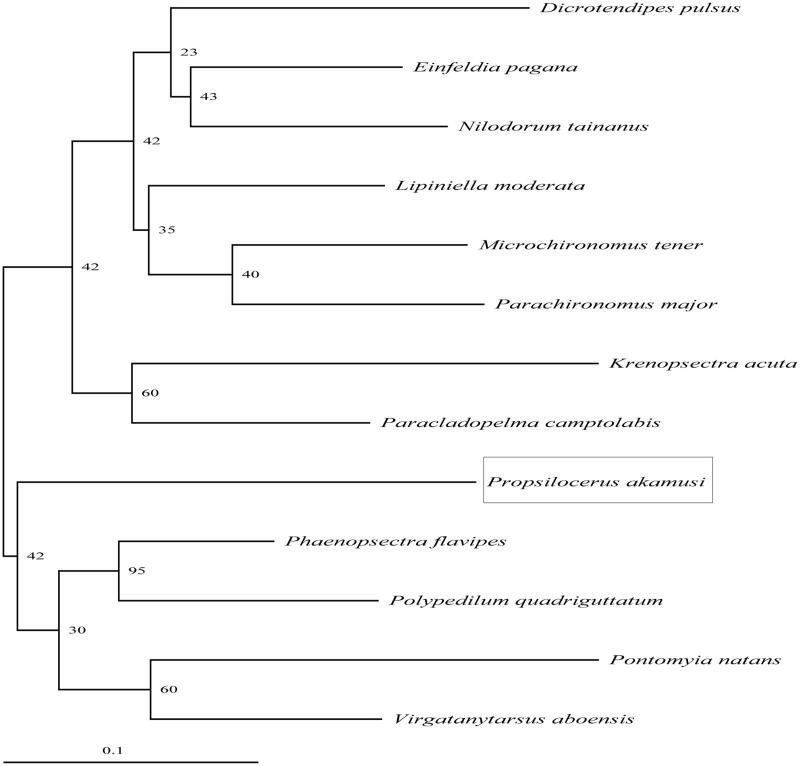
The phylogenetic relationship was estimated using the maximum likelihood method for the COI genes. Genbank accession numbers: *Beardius reissi* (HQ440878), *Baeotendipes noctivaga* (JN016825), *Dicrotendipes pulsus* (KC250805), *Einfeldia pagana* (AB838656), *Krenopsectra acuta* (FN796496), *Lauterborniella agrayloides* (HQ440927), *Lipiniella moderata* (AB838670), *Microchironomus tener* (KJ188143), *Nilodorum tainanus* (AB838672), *Phaenopsectra flavipes* (KC250831), *Paracladopelma camptolabis* (JN887074), *Polypedilum quadriguttatum* (KC250833), *Pontomyia natans* (KJ188429), *Virgatanytarsus aboensi* (AM398774), *Parachironomus major* (KC250820), and *P. akamusi* (MN566452). The numbers at the nodes are bootstrap percent probability values based on 1000 replications.
